# Dynamic Public Perceptions of the Coronavirus Disease Crisis, the Netherlands, 2020

**DOI:** 10.3201/eid2704.203328

**Published:** 2021-04

**Authors:** Marion de Vries, Liesbeth Claassen, Margreet J.M. te Wierik, Susan van den Hof, Anne E.M. Brabers, Judith D. de Jong, Danielle R.M. Timmermans, Aura Timen

**Affiliations:** National Institute for Public Health and the Environment (RIVM), Bilthoven, the Netherlands (M. de Vries, L. Claassen, M.J.M. te Wierik, S. van den Hof, A. Timen);; Netherlands Institute for Health Services Research, Utrecht, the Netherlands (A.E.M. Brabers, J.D. de Jong);; Maastricht University, Maastricht, the Netherlands (J.D. de Jong);; Amsterdam UMC, Amsterdam, the Netherlands (D.R.M. Timmermans);; Vrije Universiteit Amsterdam, Amsterdam (A. Timen)

**Keywords:** 2019 novel coronavirus disease, coronavirus disease, COVID-19, severe acute respiratory syndrome coronavirus 2, SARS-CoV-2, viruses, respiratory infections, zoonoses, perception, knowledge, attitude, trust, health behavior, communication, disease outbreaks, the Netherlands

## Abstract

A key component of outbreak control is monitoring public perceptions and public response. To determine public perceptions and public responses during the first 3 months of the coronavirus disease (COVID-19) outbreak in the Netherlands, we conducted 6 repeated surveys of ≈3,000 persons. Generalized estimating equations analyses revealed changes over time as well as differences between groups at low and high risk. Overall, respondents perceived the risks associated with COVID-19 to be considerable, were positive about the mitigation measures, trusted the information and the measures from authorities, and adopted protective measures. Substantial increases were observed in risk perceptions and self-reported protective behavior in the first weeks of the outbreak. Individual differences were based mainly on participants’ age and health condition. We recommend that authorities constantly adjust their COVID-19 communication and mitigation strategies to fit public perceptions and public responses and that they tailor the information for different groups.

Since December 2019, the world has been facing a new and severe threat to public health. Severe acute respiratory syndrome coronavirus 2 (SARS-CoV-2), which causes mild to severe respiratory illness (coronavirus disease [COVID-19]), was first found in humans in Wuhan, China (*1*). The virus spread rapidly over the world, and on March 11, 2020, the World Health Organization declared a COVID-19 pandemic (*2*). Globally, by January 23, 2021, a total of 96,877,399 cases had been confirmed, including 2,098,879 deaths (*3*). The risks associated with COVID-19 are not equally distributed; some regions (within and between countries) are more strongly affected than others, health workers are at increased risk for infection, and elderly persons with certain chronic underlying conditions and men are at increased risk for severe COVID-19 illness and death (*4*).

During the COVID-19 pandemic, countries all over the world rapidly adopted various measures to counter the spread of the virus. In the initial (containment) stage, the measures were aimed at identifying and isolating new cases. As the number of cases started to rise quickly, countries announced additional social distancing measures. Many countries undertook stringent mitigation measures, such as closing schools and restaurants, restraining domestic and foreign travel, and, for some, implementing a total lockdown of the society (*5*). For these measures to be effective, governments rely strongly on the support and compliance of the general public.

To maintain support for the protective measures for a longer period, governments need insights into the dynamics of public perceptions (regarding the risks associated with COVID-19 and the recommended protective measures) and the trust in the authorities who imposed these measures. These perceptions and trust influence the public’s compliance with the measures (*6*) and are essential indicators for public sentiments and information needs. Such insights enable optimal adaptation and tailoring of risk and crisis communication (*7*–*10*). The first publications about public perceptions of and responses to the COVID-19 pandemic are, to our knowledge, all cross-sectional and do not provide insights into the dynamics (*11*–*19*). Previous studies about the 2009 influenza A(H1N1) pandemic showed considerable changes in, among other things, perceptions of risk, trust in authorities, and self-reported protective behavior over a longer crisis period (*20*–*25*).

Our study focused on public perceptions, trust, and behavior in the first 3 months of the COVID-19 crisis in the Netherlands. Our main research question asked about the evolution of public perceptions of COVID-19, perceptions of control measures, trust in authorities, and self-reported protective behavior between the onset of the outbreak and the first relaxations of government measures. We discuss these findings in light of the epidemiologic curve of COVID-19 in the Netherlands and the government outbreak response during February–May 2020 (Figure 1). In addition, we explored differences in perceptions, trust, and self-reported behavior between groups of persons at different levels of risk. Therefore, our second research question asked whether persons differ in their perceptions of COVID-19, perceptions of the control measures, trust in authorities, and self-reported protective behavior on the basis of their age, sex, region of residence, health condition, and health sector employment.

**Figure 1:**
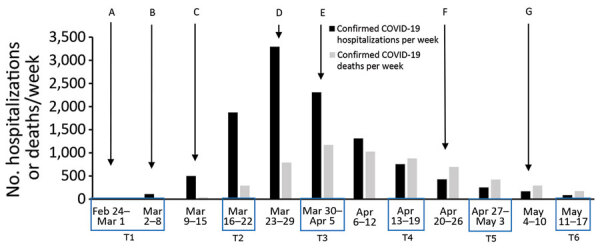
Course of COVID-19 in the Netherlands, February 24–May 17, 2020. COVID-19 hospitalizations and deaths are shown by week (data from https://www.rivm.nl/coronavirus-covid-19/grafieken). Blue boxes labeled T1–T6 along baseline indicate timing of data collection for this study. Letters indicate implementations and relaxations of COVID-19 protective measures announced by the Netherlands government in press conferences on national television (data from https://www.acaps.org/covid19-government-measures-dataset and https://www.rijksoverheid.nl/onderwerpen/coronavirus-covid-19; a selection of the measures is shown): A) All residents asked to self-isolate after receiving a COVID-19 diagnosis or if living in a household with a confirmed COVID-19 patient. B) Residents of Noord-Brabant Province (southern Netherlands) asked to self-isolate when experiencing symptoms. C) All residents experiencing symptoms asked to self-isolate, work at home as much as possible, keep distance from others. Gatherings of >100 persons prohibited; various public places closed, including (pre) schools and universities, restaurants and bars, sports clubs. D) All residents asked to stay at home as much as possible, self-quarantine when someone in the household has a fever or dyspnea. All gatherings prohibited; professions that require direct contact, such as hairdressers and masseurs, prohibited; visiting nursing homes prohibited. In some areas, mayors can prohibit groups of >3 persons who do not maintain 1.5-m distance from each other (except members of the same household). Law-enforcement allowed to fine those who do not adhere to the measures. E) All measures extended through April 28. F) Children allowed to play sports outside in groups starting April 29. Preschools and primary schools reopen (partly) starting May 11. All other measures extended through May 19. G) Starting May 11, the advice “stay at home as much as possible” replaced with the advice “avoid crowds”; gatherings up to 30 persons allowed (with 1.5-m distance); most professions that require direct contact can resume working, with extra precautions. Not indicated: Starting June 1, restaurants and bars reopen (maximum 30 persons/establishment and with 1.5-m distance); primary schools reopen (all days of the week); gatherings up to 100 persons allowed (with 1.5-m distance). COVID-19, coronavirus disease.

## Methods

### Case Study

The first COVID-19 case in the Netherlands was identified on February 27, 2020 (*26*). In the following weeks, the number of confirmed cases increased rapidly (*27*,*28*) and the number of cases between regions differed considerably. The 12 provinces in the Netherlands can be roughly divided into 4 regions: north, east, south, and west. Through May 17, the region most strongly affected by the COVID-19 outbreak was the southern region (58–63 deaths/100,000 residents), especially compared with the northern region (3–9 deaths/100,000 residents). The eastern region reported 19–31 deaths/100,000 residents and the western region 16–30 deaths/100,000 residents (*29*).

After the first case of COVID-19 was reported, the government issued various measures that increased in stringency through March 23, 2020. When a sustained decrease in the number of cases, hospitalizations, and deaths was reached (May 11, 2020), the government gradually relaxed measures (Figure 1).

### Study Population and Procedure

We conducted 6 repeated online surveys among members of the Dutch Health Care Consumer Panel (*30*). The panel consists of ≈11,000 residents of the Netherlands (>18 years of age) who had been invited to participate on the panel on the basis of a random selection of name and address data or were invited to participate by their general practitioner. The panel population is regularly renewed, and persons cannot enroll themselves without an invitation. As a result of oversampling of persons >65 years of age for other research purposes and underparticipation of persons <30 years of age, the median age of the panel population is 65 years (range 19–101 years).

The 6 repeated surveys were added to a weekly online survey that monitors influenza-like symptoms. The invitations for the first survey (time 1 [T1]) of the weekly monitor was sent to all 10,993 active panel members, who could complete the survey from February 24 through March 9, 2020. In the first survey, respondents could indicate whether they wanted to be invited for the consequent weekly surveys; those who indicated “yes” received a weekly invitation for all follow-up surveys. Participation in each survey was voluntary and did not depend on participation in previous surveys. The follow-up surveys with the variables addressed in this study were completed during March 16–23 (T2), March 30–April 5 (T3), April 14–19 (T4), April 28–May 3 (T5), and May 11–17 (T6) (Figure 1).

Before joining the panel population, potential panel members actively consented to participation and data sharing; they were informed about the purpose and content of the survey and that they could skip questions or stop participating at any time. Completing the survey, including answering questions about influenza-like symptoms, took an average of 8 minutes. The Clinical Expertise Centre at the National Institute for Public Health and the Environment (RIVM; Bilthoven, the Netherlands) determined that this research was exempt from needing further approval from an ethics research committee (reference no. LCI-451). The gathered data were analyzed and processed according the General Data Protection Regulation. More elaborate descriptions of the data collection are published elsewhere (*31*) and provided in the Appendix.

### Variables

The survey questions addressed public perceptions of COVID-19, perceptions of control measures, trust in authorities, and self-reported protective behavior. The T3 survey and subsequent surveys were supplemented with extra questions about control measures and about trust in authorities (Table 1).

**Table 1:** An overview of the survey questions and corresponding measurements used to assess dynamic public perceptions of the coronavirus disease crisis, the Netherlands, 2020*

Topic, variable	Survey question (answer category)
Perceptions of COVID-19	
Perceived probability COVID-19	In your opinion, how likely is it that you will become ill due to the new coronavirus in the next 12 months? (1. very unlikely—5. very likely)
Perceived severity of	How severe would it be to you if you develop one of the following diseases in the next 12 months? (1. Not severe at all—5. Very severe)†
Flu	Flu
COVID-19	Disease due to the novel coronavirus
Ebola	Ebola
Concerns about	Are you concerned due to the new coronavirus … (1. Not at all concerned—5. Very concerned)
Own health	About your own health?
Health of family members	About the health of your family members?
Perceptions of control measures	
Perception that sufficient measures are taken	Do you think that the Netherlands is currently taking sufficient measures to control the spread of the new coronavirus? (1. Certainly not—5. Certainly yes)‡
Perceptions of the recommended measures§	Below there are several statements about the measures advised by the government to control the spread of the coronavirus. Please state what you think about these statements. (1. Certainly not—5. Certainly yes)
Measures are effective	I think the recommended measures help to control the spread of the coronavirus
Most others adhere to measures	Most people close to me adhere to the recommended measures.
Difficult to adhere to measures	I find it difficult to adhere to the recommended measures.
Trust in authorities	
Trust in information from the National Institute for Public Health and the Environment (RIVM)§	How much trust do you have in the information from the National Institute for Public Health and the Environment (RIVM) about the new coronavirus? (1. No trust—5. A lot of trust)
Trust in government measures§	How much trust do you have in the measures that the government is taking to control the spread of the new corona virus? (1. No trust—5. A lot of trust)
Self-reported protective behavior	
Adopted protective measures	Have you taken measures to protect yourself or your family members from the new coronavirus? (1. No / 2. Yes, namely…)
Adherence to recommended measures§	Do you adhere to the guidelines advised by the government to control the spread of the new coronavirus? (1. Yes / 2. Partly / 3. No / 4. Don’t know)¶

*COVID-19, coronavirus disease; Flu, influenza; T1–T6, surveys 1–6. †Adapted from previous studies on public responses to influenza A(H1N1) (*25*) and Ebola (*32*) to allow for comparison with previous crises and to place the perceived severity of COVID-19 into context with other diseases. These are, from an expert’s perspective, less severe (flu) and more severe (Ebola) infectious diseases than COVID-19. ‡Formulated in T1 and T2 as “Do you think that the Netherlands is currently taking sufficient measures to prevent the spread of the new coronavirus?” §Not assessed at T1 and T2. ¶The answer categories “partly,” “no,” and “don’t know” were merged into 1 value next to the value “yes” because of low response frequencies to the categories “no” and “don’t know.”

The factors that put persons at increased risk for COVID-19 were operationalized as sex (male/female), age group (<50, 50–69, or >70 years of age), region of residence (north, east, west, south; variable determined on the basis of postal codes), employment in healthcare (assessed at T1 with the question “Do you currently work in the healthcare sector?: no/yes”), and underlying health condition [assessed at T1 with the question “Please mark the disease(s) or condition(s) you have below. (Multiple answers possible): A) chronic respiratory disease; B) serious heart disease or myocardial infarction; C) diabetes; D) an allergy such as hay fever, dust mite allergy, or pet allergy; E) other long-term or chronic condition, namely: … F). I don't have any diseases or conditions).”]. The last variable was recoded as “underlying health condition” if respondents answered A, B, C, or E; all others were coded as “no underlying health condition.”

### Analyses

We computed descriptive statistics for each variable in T1–T6 (Table 1). To study changes over time and differences between persons in these variables, we performed generalized estimating equation (GEE) analyses (with exchangeable correlation matrix). We performed linear (for dependent variables with a 5-point Likert scale) and logistic (for dependent variables with binary outcomes) GEE analyses. The independent variables were time (T1–T6), sex, age group, region of residence, underlying health condition, and employment in healthcare. All GEE analyses were controlled for education level and income. To observe all changes between the subsequent waves, we repeated all GEE analyses with different reference groups for time (T1, T3, and T5). We excluded from analysis respondents who participated in T1 but did not consent to be invited to participate in the follow-up surveys.

## Results

### Study Population

Of the 10,993 persons invited to participate, 4,325 (39%) completed the first survey. Of note, 2,052 respondents completed the first survey before February 27, 2020 (when the first COVID-19 case in the Netherlands was confirmed). A total of 3,268 (30%) consented to be invited for the follow-up surveys, of which 2,592 participated in T2 (79%), 2,710 in T3 (83%), 2,726 in T4 (83%), 2,654 in T5 (81%), and 2,705 in T6 (83%) (*33*) (Table 2; Appendix).

**Table 2:** Characteristics of respondents to the first survey who consented to participation and were invited to participate in successive surveys used to assess dynamic public perceptions of the coronavirus disease crisis, the Netherlands, 2020*

Characteristic	No. (%)
Sex	
M	1,644 (50)
F	1,624 (50)
Age, y	
<30	24 (1)
30–49	530 (16)
50–69	1,220 (37)
>70	1,494 (46)
Education level*	
Low	336 (10)
Middle	1,528 (47)
High	1,352 (41)
Unknown	52 (2)
Monthly household income, €	
<1,750	661 (20)
1,750–2,700	1,078 (33)
>2,700	1,399 (43)
Unknown	130 (4)
Region of residence	
North	539 (16)
East	738 (23)
South	655 (20)
West	1,320 (40)
Unknown	16 (1)
Underlying health condition	
Present	1,567 (48)
Absent	1,649 (50)
Unknown	52 (2)
Work in healthcare	
Yes	359 (11)
No	2,886 (88)
Unknown	23 (1)
Total	3,268 (100)

*Operationalization (*33*).

### Perceptions of COVID-19

**Figure 2:**
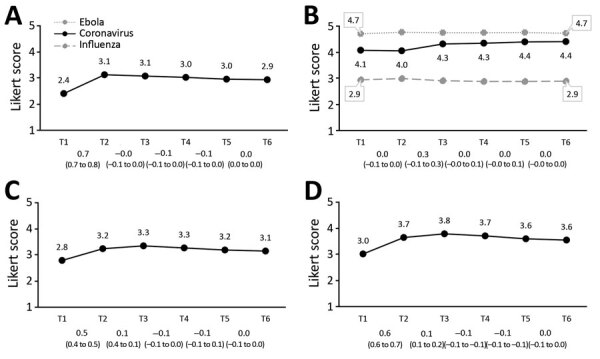
Perceptions of COVID-19 in the Netherlands. A) Perceived probability of COVID-19; B) perceived severity of influenza, coronavirus disease, Ebola; C) concerns about own heath; D) concerns about health of family members. Mean values per survey are shown above the graph line. Note that the 95% CIs around the mean estimates could not be shown on the figure because the 95% CIs are very close to the mean estimates (upper values of <mean + 0.1 and lower values of >mean – 0.1). All 95% CIs around the mean estimates are shown in Appendix Table 2. Changes between subsequent surveys, based on generalized estimating equation analyses, are shown below the baselines as β and 95% CIs. The coefficients and 95% CIs shown in Figure 3, panel B, are generalized estimating equation results with perceived severity of coronavirus disease as the dependent variable. COVID-19, coronavirus disease.

Overall, respondents perceived acquiring COVID-19 as probable and considerably severe (Figure 2). The perceived severity of COVID-19 was more similar to that of Ebola than that of influenza. Concerns about their own health were substantial, and concerns about the health of family members were even more so.

The most considerable change in the perceptions of COVID-19 was seen between T1 and T2; the mean perceived probability of COVID-19, concerns about one’s own health, and concerns about family members increased considerably. Perceived severity of COVID-19 increased (significantly) only between T2 and T3. Between T3 and T6, perceptions of COVID-19 were largely stable, except for a slight but significant decrease in concerns.

### Perceptions of Control Measures

**Figure 3:**
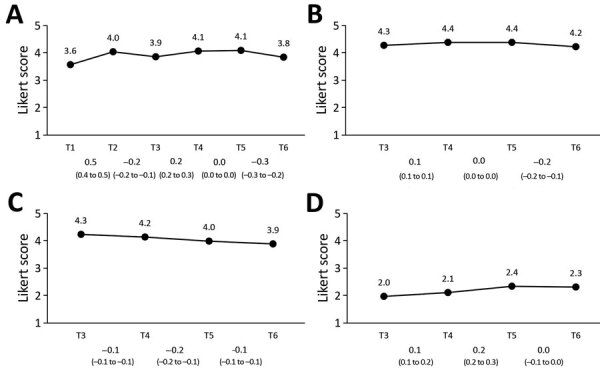
Perceptions of coronavirus disease control measures in the Netherlands. A) Sufficient measures are taken; B) measures are effective; C) most others adhere to measures; D) difficult to adhere to measures. Mean values per survey are shown above the graph line. Note that the 95% CIs around the mean estimates could not be shown in the figure because the 95% CIs are very close to the mean estimates (upper values of <mean + 0.1 and lower values of >mean – 0.1). All 95% CIs around the mean estimates are shown in Appendix Table 2. Changes between subsequent surveys, based on generalized estimating equation analyses, are shown below the baselines as β and 95% CIs.

Overall, respondents thought that the Netherlands undertook sufficient measures to control the spread of COVID-19, perceived the recommended measures as effective, thought that most others adhered to the measures, and did not perceive adhering to the measures as difficult (Figure 3). The perception that the Netherlands was taking sufficient measures changed nonlinearly between T1 and T6. Perception of measure effectiveness followed a pattern similar to the perception that sufficient measures were taken; it slightly increased between T3 and T4 and slightly decreased between T5 and T6. The perception that most others adhere to the measures decreased gradually between T3 and T6, and the perceived difficulty of adhering to the measures increased slightly between T3 and T5.

### Trust in Authorities

**Figure 4:**
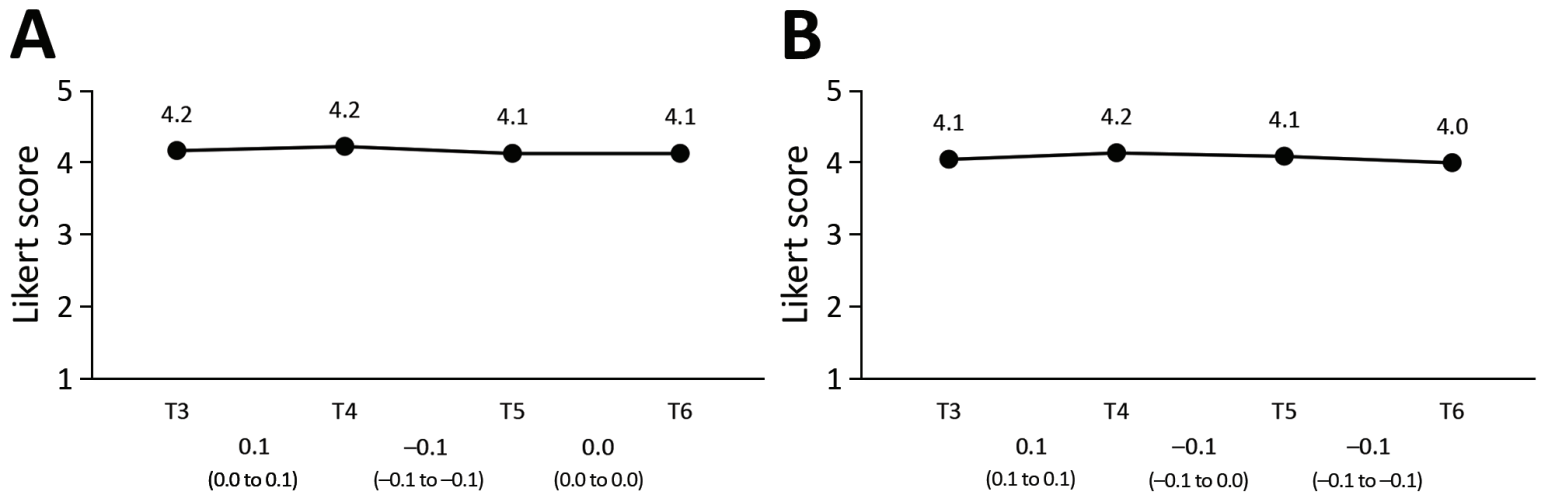
Trust in authorities in the Netherlands. A) Trust information from National Institute for Public Health and the Environment (RIVM), Bilthoven, the Netherlands; B) trust government measures. Mean values per survey are shown above the graph line. Note that the 95% CIs around the mean estimates could not be shown in the figure because the 95% CIs are very close to the mean estimates (upper values of <mean + 0.1 and lower values of >mean – 0.1). All 95% CIs around the mean estimates are shown in Appendix Table 2. Changes between subsequent surveys, based on generalized estimating equation analyses, are shown below the baselines as β and 95% CIs.

Overall, trust in the information from RIVM and in the measures taken by the government was fairly high (Figure 4). A slight decrease in trust in the information from RIVM was observed between T4 and T5. Trust in the measures from the government slightly increased between T3 and T4 and slightly decreased between T5 and T6.

### Self-Reported Protective Behavior

**Figure 5:**
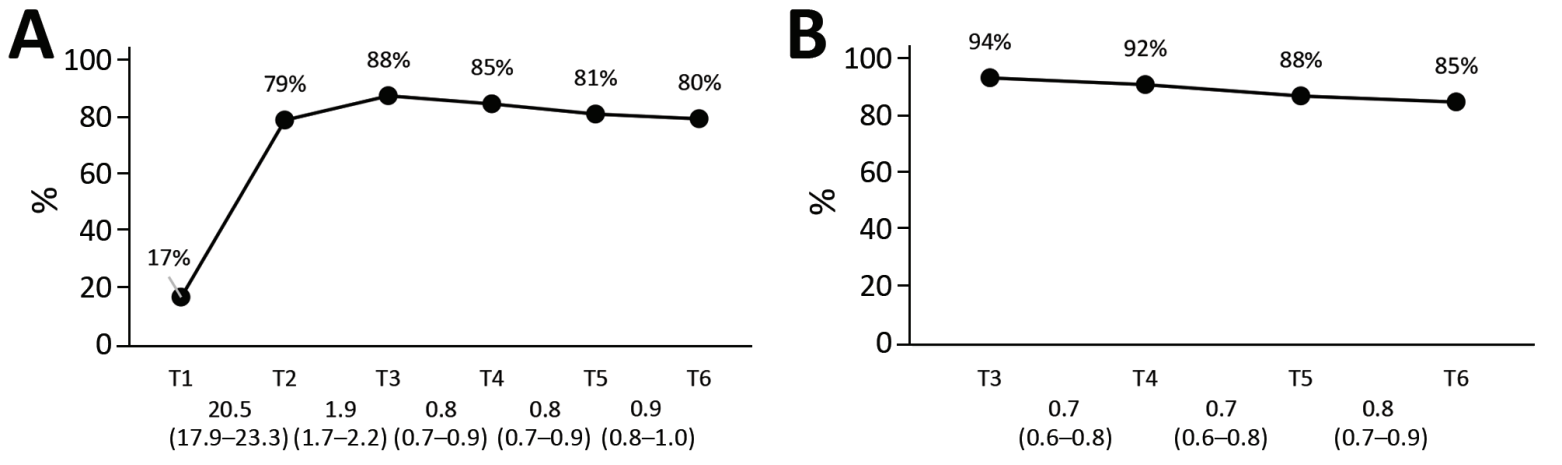
Self-reported coronavirus disease protective behavior in the Netherlands. A) Self-reported protective measures taken; B) self-reported adherence to guidelines. Mean values per survey are shown above the graph line. Note that the 95% CIs around the mean estimates could not be shown in the figure because the 95% CIs are very close to the mean estimates (upper values of <mean + 0.1 and lower values of >mean – 0.1). All 95% CIs around the mean estimates are shown in Appendix Table 2. Changes between subsequent surveys, based on generalized estimating equation analyses, are shown below the baselines as odds ratios and 95% CIs.

From T1 through T2, the proportion of respondents who indicated that they took measures to protect themselves or their family members against SARS-CoV-2 increased drastically, from 17% to 79% (Figure 5). From T2 through T3, this percentage increased further, to 88%, and consequently decreased to 80% at T6. Likewise, from T3 through T6, the proportion of respondents who indicated that they (fully) adhered to the recommended guidelines declined gradually from 94% to 85%.

### Differences Based on Risk Factors

The most notable differences between persons in terms of perceptions (Tables 3, 4), trust in authorities (Table 5), and self-reported protective behavior (Table 6) were based on age. Compared with persons <50 years of age, those 50­–69 and >70 years of age perceived acquisition of COVID-19 as being less probable and COVID-19 as more severe and were more concerned about their own health. In addition, respondents >70 years of age were also more likely to perceive the government’s measures as sufficient, effective, and adhered to by most others and were less likely to perceive adhering to the measures as difficult. That difference in perceived difficulty was also observed for those 50–69 compared with those <50 years of age. Participants >70 years of age also experienced more trust in authorities and were more likely to adhere to the guidelines.

**Table 3:** Differences in perceptions of COVID-19 based on sex, age, region of residence, health condition, and healthcare employment determined in assessment of dynamic public perceptions of the coronavirus disease crisis, the Netherlands, 2020*

Independent variable	Perceived probability of COVID-19, β (95% CI)	Perceived severity of COVID-19, β (95% CI)	Concerns about own health, β (95% CI)	Concerns about health of family members, β (95% CI)
Female vs. male	**0.1 (0.1 to 0.1)**	**0.1 (0.1 to 0.2)**	0.1 (0 to 0.1)	**0.2 (0.1 to 0.2)**
Age, y				
>70 vs. <50	**–0.3 (–0.4 to –0.3)**	**0.6 (0.5 to 0.7)**	**0.4 (0.3 to 0.4)**	0 (−0.1 to 0)
50–69 vs. <50	**–0.2 (–0.3 to –0.2)**	**0.4 (0.3 to 0.4)**	**0.2 (0.1 to 0.2)**	−0.1 (−0.2 to 0)
Region				
Southern vs. northern	0 (−0.1 to 0.1)	0.1 (0 to 0.2)	**0.1 (0.1 to 0.2)**	0.1 (0 to 0.2)
Western vs. northern	0 (−0.1 to 0.1)	0.1 (0 to 0.1)	0.1 (0 to 0.1)	0 (0 to 0.1)
Eastern vs. northern	0 (0 to 0.1)	0 (0 to 0.1)	0 (−0.1 to 0.1)	0 (−0.1 to 0.1)
Health condition vs. no health condition	**0.2 (0.1 to 0.2)**	**0.1 (0.1 to 0.2)**	**0.4 (0.3 to 0.4)**	**0.2 (0.2 to 0.3)**
Work in healthcare vs. not in healthcare	**0.1 (0.1 to 0.2)**	−0.1 (−0.2 to 0)	−0.1 (−0.2 to 0)	0 (−0.1 to 0.1)

*Survey questions shown in Table 1. Boldface indicates 95% CIs that do not include 0. COVID-19, coronavirus disease.

**Table 4:** Differences in perceptions of control measures based on sex, age, region of residence, health condition, and healthcare employment determined in assessment of dynamic public perceptions of the coronavirus disease crisis, the Netherlands, 2020*

Independent variable	Sufficient measures are taken, β (95% CI)	Measures are effective, β (95% CI)	Most others adhere to measures, β (95% CI)	Difficult to adhere to measures, β (95% CI)
Female vs. male	**0.1 (0.1 to 0.2)**	**0.1 (0.1 to 0.2)**	**0.2 (0.2 to 0.2)**	−0.1 (−0.1 to 0)
Age, y				
>70 vs. <50	**0.2 (0.2 to 0.3)**	**0.2 (0.1 to 0.2)**	**0.2 (0.2 to 0.3)**	**–0.2 (–0.3 to –0.1)**
50–69 vs. <50	0.1 (0 to 0.2)	0.1 (0 to 0.1)	0.1 (0 to 0.2)	**–0.3 (–0.4 to 0.2)**
Region				
Southern vs. northern	0 (−0.1 to 0.1)	0 (−0.1 to 0.1)	−0.1 (−0.1 to 0)	0 (−0.1 to 0.1)
Western vs. northern	0 (−0.1 to 0.1)	0 (−0.1 to 0.1)	−0.1 (−0.1 to 0)	0 (−0.1 to 0.1)
Eastern vs. northern	0.1 (0 to 0.2)	0.1 (0 to 0 1)	0 (−0.1 to 0)	0 (−0.1 to 0.1)
Health condition vs. no health condition	**–0.2 (–0.2 to –0.1)**	−0.1 (−0.1 to 0)	−0.1 (−0.1 to 0)	0 (−0.1 to 0.1)
Work in healthcare vs. not in healthcare	0.1 (0 to 0.1)	0 (−0.1 to 0.1)	0 (−0.1 to 0.1)	0.1 (0 to 0.2)

*Actual survey questions shown in Table 1. Boldface indicates 95% CIs that do not include 0. COVID-19, coronavirus disease.

**Table 5:** Differences in trust in authorities based on sex, age, region of residence, health condition, and healthcare employment determined in assessment of dynamic public perceptions of the coronavirus disease crisis, the Netherlands, 2020*

Independent variable	Trust RIVM information, β (95% CI)	Trust government measures, β (95% CI)
Female vs. male	0.1 (0 to 0.1)	0.1 (0 to 0.2)
Age, y		
>70 vs. <50	**0.3 (0.2 to 0.4)**	**0.3 (0.2 to 0.4)**
50–69 vs. <50	0.1 (0 to 0.2)	0.1 (0 to 0.2)
Region		
Southern vs. northern	0 (−0.1 to 0.1)	0.1 (0 to 0.1)
Western vs. northern	0.1 (0 to 0.1)	0 (0 to 0.1)
Eastern vs. northern	**0.2 (0.1 to 0.2)**	**0.1 (0.1 to 0.2)**
Health condition vs. no health condition	**–0.1 (–0.2 to –0.1)**	**–0.1 (–0.2 to –0.1)**
Work in healthcare vs. not in healthcare	0.1 (0 to 0.2)	0 (−0.1 to 0.1)

*Actual survey questions shown in Table 1. Boldface indicates 95% CIs that do not include 0. RIVM, National Institute for Public Health and the Environment.

**Table 6:** Differences in self-reported protective behavior based on sex, age, region of residence, health condition, and healthcare employment determined in assessment of dynamic public perceptions of the coronavirus disease crisis, the Netherlands, 2020*

Independent variable	Self-reported protective measures taken, odds ratio (95% CI)	Self-reported adherence to guidelines, odds ratio (95% CI)
Female vs. male	**1.8 (1.6 to 2.1)**	1.2 (1.0 to 1.5)
Age, y		
>70 vs. <50	1.2 (1.0 to 1.5)	**1.7 (1.3 to 2.2)**
50–69 vs. <50	1.1 (0.9 to 1.4)	1.2 (0.9 to 1.6)
Region		
Southern vs. northern	1.1 (0.9 to 1.4)	0.9 (0.6 to 1.2)
Western vs. northern	0.9 (0.7 to 1.1)	1.0 (0.8 to 1.3)
Eastern vs. northern	1.1 (0.9 to 1.3)	1.2 (0.9 to 1.7)
Health condition vs. no health condition	**1.3 (1.1 to 1.4)**	1.0 (0.8 to 1.2)
Work in healthcare vs. not in healthcare	0.9 (0.7 to 1.2)	0.8 (0.6 to 1.0)

*Actual survey questions shown in Table 1. Boldface indicates 95% CIs that do not include 1.0.

We observed several differences between respondents with and without an underlying health condition. Respondents with an underlying health condition perceived acquisition of COVID-19 as being more probable and COVID-19 as being more severe, and they were more concerned about their own health and that of family members. These respondents also perceived that measures taken were less sufficient, had less trust in authorities, and were slightly more likely to have adopted protective measures.

Some additional small differences were observed on the basis of sex, region, and employment. Women perceived the probability of acquiring and severity of COVD-19 as being somewhat greater than did men and were slightly more concerned about family members. In addition, women were somewhat more positive about the measures (sufficient, effective, and adhered to by others) and were more likely to have adopted protective measures. Compared with residents from the northern region of the Netherlands, residents from the southern region were slightly more concerned about their own health, and residents from the eastern region had somewhat more trust in authorities. The only difference based on employment in the healthcare sector was seen in perceived probability of a SARS-CoV-2 infection (slightly higher among healthcare workers).

## Discussion

Our results suggest that during the first wave of COVID-19, persons in the Netherlands generally perceived the risks posed by COVID-19 as considerable, were positive about the measures taken by the government to control the spread of COVID-19, trusted the information and the measures from the authorities in charge of the control policy, and adopted protective behavior. Public perceptions and behavior changed between the onset of the crisis and the initial relaxation of measures, particularly in the first phase of the outbreak. Differences between persons were mostly seen on the basis of age and underlying health conditions.

The changes in public perceptions, trust, and behavior need to be interpreted in light of the rapid developments in the epidemiologic curve of COVID-19 and the outbreak response during this study (Figure 1). After the first confirmed COVID-19 case (February 27, 2020), the outbreak unfolded rapidly and stringent control measures were issued during live press conferences on national television (March 12 and 15). These developments are probably reflected in the observed increases in the respondents’ perceived probability of acquiring COVID-19, concerns, and self-reported protective behavior in this period. Up to the end of March/beginning of April, the number of COVID-19 cases rose rapidly, as did the number of hospitalizations, intensive care unit admissions, and deaths. The increased visibility of severe COVID-19 illness and death during this period might have increased perceptions of severity (which had remained stable in the first weeks).

As the number of cases, hospitalizations, intensive care unit admissions, and deaths gradually declined at the beginning of May, the government announced gradual relaxations of the control measures. During this period, the number of respondents who reported having taken protective measures and adhered to the recommended guidelines declined. This change in protective behavior is not likely to be explained by a change in risk perception, perception of the efficacy of the measures, or trust in authorities (factors shown to influence behavior during disease outbreaks [*6*,*9*,*13*]) because these factors were stable during this period. This change in behavior might be partly explained by a decrease in the public’s perceived self-efficacy (*6*,*34*) because during this period we observed an increase in the public’s perceived difficulty of adhering to the measures.

More recent research in the Netherlands has shown that in the months after our study, persons perceived it to be increasingly difficult to adhere to several of the control measures (*35*). Although the perceived difficulty of not shaking hands and practicing proper handwashing remained relatively stable, the perceived difficulty of maintaining a 1.5-m distance from others increased considerably from mid-April through mid-July 2020. Another study also found fairly high compliance with hygiene measures during the COVID-19 pandemic, along with limited compliance on social distancing measures (*36*). This finding might be explained by the assumed negative effect of social distancing on mental health and loneliness (*37*–*39*). It is understandable that persons find it (increasingly) hard to be apart from others, specifically from their loved ones. Other factors, such as more practical barriers (e.g., difficult to keep distance in small corridors in the supermarket) (*40*) and perceived social norms (*41*), might also play a role.

Trust in the information and the measures from authorities was relatively high and stable throughout the first wave of the COVID-19 crisis. Other studies from New Zealand (*42*) and South Korea (*43*) have shown increased trust in government during the spring of 2020 compared with earlier years, which the authors attributed to the decisive and rapid governmental crisis response. A study in the United Kingdom suggests that trust can also rapidly decline, which was observed after government announcements to relax lockdown measures and news of misconduct by a high government official (*44*). Of note, recent research has also shown a decrease in public trust in the government’s approach to the COVID-19 crisis in the Netherlands from the end of May through the beginning of October (*45*). Whether this decreased trust is explained by relaxations of measures or other events/processes needs further investigation.

In our study, the differences in perceptions, trust, and self-reported behavior between subgroups were rather small. Overall, the largest observed differences were based on age and health condition. Older persons perceived COVID-19 as more severe and had more concerns about their own health than did younger persons. At the same time, older persons perceived the probability of their getting infected with the virus to be lower. A similar result was found in an earlier study on COVID-19 risk perceptions, which showed increased perceived risk for death among elderly persons but lower perceived risk for infection (*14*). An explanation for the lower perceived risk is that older persons might have adopted more stringent social distancing measures than younger persons and therefore perceived their risk for infection as being smaller. In formal communications, maintaining strict social distancing was recommended for persons >70 years of age, and it was recommended that everyone avoid visiting elderly persons (*46*). Respondents with a chronic health condition also perceived their risk of becoming infected to be more probable and the infection to be more severe, and they were more concerned than those with no underlying health condition.

In line with risk-perception literature and previous research on behavior during disease outbreaks (*6*), we also found small differences on the basis of sex. Although the risk for severe COVID-19 illness is higher for men (*4*), women in our study indicated slightly higher risk perceptions and were more likely to adopt measures to protect themselves and their family. Despite the considerable differences in infection rates between the different regions in the Netherlands and the increased risk to healthcare workers (*4*) and in contrast to previous perception study findings (*15*,*19*), we found few differences between persons on the basis of region of residence and healthcare employment.

One study limitation is that the study sample is not perfectly representative of the population of the Netherlands at large; specifically, our study included few respondents <30 years of age. Another limitation is that our operationalization of the variable “underlying health condition” includes all self-reported chronic or long-term conditions (except for allergies). At the start of this study, little was known about the specific underlying health conditions associated with increased risk for COVID-19, and these underlying conditions have therefore not been separately added to the survey as answer categories. In addition, the behaviors reported in our study are self-reported and might be subject to social desirability bias.

Our findings emphasize the need to monitor public perceptions and public responses among different groups during crises because these perceptions can change considerably over time and can differ among persons. Such insights are needed to be able to respond to changes in public perceptions and public responses with timely and accurate risk and crisis communication. To maintain public compliance with protective measures during the COVID-19 crisis, we also need to understand why persons struggle with adhering to these measures and what they need to help them overcome these difficulties. Consulting and collaborating with communities to understand their difficulties and needs during this unprecedented crisis is pivotal. When differences between perceptions, responses, and needs in certain groups are large (e.g., between younger and older persons), targeting or tailoring information to specific groups is advisable. Such group-targeted information should be well-adapted to common views in a specific group and should reach the group through various accessible channels (e.g., social media or postal mail) or intermediaries (e.g., schoolteachers, general practitioners).

Appendix Supplementary methods and results for study of dynamic public perceptions of the coronavirus disease crisis, the Netherlands, 2020.
