# Supplementary material for: Dynamic Public Perceptions of the Coronavirus Disease Crisis, the Netherlands, 2020
Source: Emerg Infect Dis. 2021 Apr;27(4):1098–109. doi: 10.3201/eid2704.203328 (PMC8007322; doi:10.3201/eid2704.203328)
Supplement: Appendix — Supplementary methods and results for study of dynamic public perceptions of the coronavirus disease crisis, the Netherlands, 2020. [file 20-3328-Techapp-s1.pdf]

Article DOI: https://doi.org/10.3201/eid2704.203328

Dynamic Public Perceptions of the Coronavirus Disease Crisis, the Netherlands, 2020

Appendix

A description of the Dutch Healthcare Consumer Panel and the data collection

The Dutch Healthcare Consumer Panel

The survey data reported on in this study was collected among members of the Dutch Health Care Consumer Panel (*1*). This panel consists of ≈11,000 Dutch residents (aged 18+) who have agreed to answer surveys about health related topics on a regular basis. The panel members are recruited via two routes, namely a) via a postal invitation based on a random selection of name and address data and b) via general practices. These two ways of recruitment are described more elaborately in the sections below.

It is not possible for people to enroll in the panel without invitation. In addition, the panel is regularly renewed; panel members who have been participating for a longer period of time are replaced with new members. The impossibility to join the Dutch Healthcare Consumer Panel without invitation, and the renewal of the panel, makes this panel, assumable, more representative to the larger population than the many commercial ‘opt-in’ panels used for research (*2*,*3*). The impossibility to join the panel without invitation prevents, to some extent, that respondents with a strong interest in survey participation and healthcare topics are overrepresented in the panel. In addition, the renewal of the panel prevents learning effects in survey participation and questionnaire ‘fatigue’.

Within the panel people aged >65 years are overrepresented. This is because during several recruitments, extra elderly (>65 years) were recruited for a long-term study within the panel about social participation among the elderly.

Recruitment based on names and addresses

Part of the panel consists of members who have been invited based on a random selection of name and address data. Lists with a random selection of names and addresses are bought from a third party. In addition, if members with certain demographics are underrepresented in the panel, invitations are based on a purposive selection of name and address data.

Recruitment via general practices

Part of the panel consists of members who have been recruited via general practices that participate in the Nivel Primary Care Database (*4*). Nivel, the organization behind the Dutch Health Care Consumer Panel, collaborates with general practices in the Netherlands for systemic healthcare research. Thirteen of these general practices located in different regions in the Netherlands contribute to the Dutch Health Care Consumer Panel by sending their list with patient addresses to a trusted third party. This trusted third party removes all persons aged younger than 18 years and older than 85 years from the list, and consequently selects one person from each individual address. These persons consequently receive a postal invitation to participate in the Dutch Health Care Consumer Panel. The panel members who were recruited via this route were asked for (voluntary) consent to combine their survey data with data registered at their general practitioner.

The data collection for the current study

Between 24 February and 17 May 2020, a weekly survey was send to members of the Dutch Health Care Consumer Panel to assess flu-like symptoms during the first wave of the COVID-19 crisis in the Netherlands (*5*). The survey questions regarding public perceptions and responses to the COVID-19 crisis were added to six of these weekly surveys. These surveys were collected between 24 February – 9 March 2020 (T1), 16 – 23 March (T2), 30 March – 5 April (T3), 14 – 19 April (T4), 28 April – 3 May (T5) and 11 - 17 May (T6). All survey rounds were open for participation for approximately 1 week (Monday 2 p.m. to Monday 10 a.m.), except for the first survey round which was open for 2 weeks. The T3 and T4 survey rounds were opened on a Tuesday instead of Monday because of national holidays on the Monday in those weeks. We planned to collect data on public perceptions and responses every 3 weeks, as we assumed that this would be an appropriate time interval to monitor changes in perceptions and responses.

However, after T2 data collection, because of the rapid developments in the COVID-19 crisis, we chose to collect data on perceptions and responses every 2 weeks instead.

Recruitment of respondents

All active panel members (N = 10.993) were invited for participation to the first survey. The panel members were either invited via post (N = 4295) in the week of 24 February 2020 or via e-mail (N = 6698) on 24 February 2020, depending on which route they had registered as their preferred route for panel participation. Members who receive invitations via e-mail have indicated to prefer participating in online surveys, whereas members who receive invitations via post have indicated to prefer offline survey participation. Both in the e-mail invitation and in the postal invitation, a weblink was provided to participate in an online survey. Because the survey was administered weekly, offline survey participation was not deemed possible. The first survey was open for 2 weeks (24 February - 9 March). Panel members who were invited via e-mail were sent a reminder on 28 February, panel members who were invited via post did not receive a reminder. All participants to the first survey were asked whether they consented to be invited for a follow-up weekly online survey.

Response rate

The Appendix Figure provides an overview of the response rate for the first survey per invitation route (post / e-mail), and the proportion of respondents who consented to be invited for the follow-up weekly surveys. In total, 4325 from the 10.993 invited panel members completed the first survey (39%). The response to the first survey was considerably higher among those who received an invitation via e-mail (46%) than among those who received an invitation via post (20%). This is likely explained by the fact that those panel members invited by post do generally only participate in offline surveys and not in online surveys.

In total, 3268 from the 4325 respondents consented to be weekly invited for a follow-up survey. Each of these 3268 respondents consequently received a weekly e-mail with an invitation for participation to the weekly survey. Of these 3268 respondents, 2592 respondents participated at T2 (79%), 2710 at T3 (83%), 2726 at T4 (83%), 2654 at T5 (81%), and 2705 at T6 (83%). If we take the 10.993 active panel members as the nominator, the response rate was 39% at T1 and 24%/25% at T2 to T6.

Description of the survey population

A description of the study population at each survey wave is shown in Table 1. This figure also shows data on age, sex, education level and region of residence for the Dutch population at large. Our study population differs to some extent from the Dutch population: Our study population underrepresents people of younger age and people with a low education level, whereas it overrepresents people of older age and people with a high education level. In terms of the distribution men/women and region of residence, our study population is fairly similar to the Dutch population at large.

References

1. 1. Brabers AEM, Reitsema-van Rooijen M, de Jong JD. Consumentenpanel Gezondheidszorg: Basisrapport met informatie over het panel (2015). [cited 2021 Jan 13]. https://www.nivel.nl/nl/publicatie/consumentenpanel-gezondheidszorg-basisrapport-met-informatie-over-het-panel-2015

2. Baker R, Brick JM, Couper MP, Courtright M, Dennis JM, Dillman D, et al. Research synthesis: AAPOR report on online panels. Public Opin Q. 2010;74:711–81. https://doi.org/10.1093/poq/nfq048

3. Brüggen E, van den Brakel J, Krosnick J. Establishing the accuracy of online panels for survey research. Statistics Netherlands;2016.

4. Nivel. nivel primary care database [cited 2020 Nov 27]. https://www.nivel.nl/en/nivel-primary-care-database.

5. Brabers A, Meijer M, Hooiveld M. de Jong, J., *Monitor griepachtige klachten en uitbraak van het coronavirus: het perspectief van de burger. Methodologische verantwoording.* 2020, Nivel: Utrecht, The Netherlands.

Appendix Table 1. Description of the study population at each survey wave. For the first survey wave (24 Feb – 1 March) only the 3268 respondents who agreed to be invited for the follow-up surveys are shown.

Variable	Survey wave	Feb 24–Mar 1		Mar 16–23		Mar 30–Apr 5		Apr 13–19		Apr 29 –May 3		May 11–17		Dutch population at large* - %
	Category	No.	%	No.	%	No.	%	No.	%	No.	%	No.	%	
Sex	Men	1644	50%	1269	49%	1337	49%	1352	50%	1339	50%	1362	50%	50%
	Women	1624	50%	1323	51%	1373	51%	1374	50%	1315	50%	1343	50%	50%
Age in years	18–29	24	1%	16	1%	18	1%	17	1%	13	0%	12	0%	19%
	30–49	530	16%	437	17%	426	16%	427	16%	391	15%	388	14%	31%
	50–69	1220	37%	1000	39%	1035	38%	1039	38%	1010	38%	1029	38%	33%
	>70	1494	46%	1139	44%	1231	45%	1243	46%	1240	47%	1276	47%	17%
Education level	Low	336	10%	246	9%	261	10%	265	10%	253	10%	271	10%	32%
	Middle	1528	47%	1185	46%	1255	46%	1260	46%	1243	47%	1250	46%	39%
	High	1352	41%	1124	43%	1153	43%	1162	43%	1120	42%	1149	42%	29%
	Unknown	52	52%	37	1%	41	2%	39	1%	38	1%	35	1%	
Region of residence	North	539	16%	427	16%	435	16%	442	16%	428	16%	432	16%	10%
	East	738	23%	575	22%	625	23%	628	23%	607	23%	617	23%	21%
	West	1320	40%	1059	41%	1092	40%	1087	40%	1059	40%	1091	40%	48%
	South	655	20%	519	20%	544	20%	554	20%	544	20%	551	20%	21%
	Unknown	16	0%	12	0%	14	1%	15	1%	16	1%	14	1%	
Monthly net household income in euros	<1750	661	20%	511	20%	520	19%	522	19%	516	19%	524	19%	
	1750 - 2700	1078	33%	846	33%	897	33%	896	33%	876	33%	889	33%	
	>2700	1399	43%	1140	44%	1190	44%	1202	44%	1164	44%	1190	44%	
	Unknown	130	4%	95	4%	103	4%	106	4%	98	4%	102	4%	
Underlying health condition	Present	1567	48%	1302	50%	1371	51%	1383	51%	1342	51%	1369	51%	
	Absent	1649	50%	1255	48%	1300	48%	1301	48%	1270	48%	1294	48%	
	Unknown	52	52%	35	1%	39	1%	42	2%	42	2%	42	2%	
Work in healthcare	Yes	359	11%	292	11%	299	11%	310	11%	288	11%	293	11%	
	No	2886	88%	2285	88%	2392	88%	2396	88%	2346	88%	2392	88%	
	Unknown	23	1%	15	1%	19	1%	20	1%	20	1%	20	1%	
Total		3268	100%	2592	100%	2710	100%	2726	100%	2654	100%	2705	100%	

* These data are retrieved from the Dutch Central Bureau for Statistics (https://www.opendata.cbs.nl). The figures for sex, age and region are based on data from 1 January 2020, the figures for educational level are based on data from 2018. The percentages for sex and region are based on data from all Dutch residents. The percentages for age are based on the Dutch population aged 18 y and older. The percentages for education level are based on the Dutch population aged 15 y and older.

Appendix Table 2. Means (for variables measured on a 5-point Likert scale) and percentages (for binary variables) with 95% confidence intervals

	Survey wave	Feb 24–Mar 1			Mar 16–23			Mar 30–Apr 5			Apr 13–19			Apr 29 –May 3			May 11–17		
Survey topic	Variable	M	95% CI		M	95% CI		M	95% CI		M	95% CI		M	95% C		M	95% CI	
Perceptions of COVID-19	Perceived probability COVID-19	2.41	2.38	2.45	3.13	3.10	3.17	3.08	3.05	3.11	3.03	3.00	3.06	2.96	2.93	2.99	2.94	2.91	2.98
	Perceived severity COVID-19	4.06	4.03	4.09	4.04	4.01	4.08	4.30	4.27	4.34	4.33	4.30	4.36	4.38	4.35	4.41	4.39	4.36	4.43
	Perceived severity flu*	2.93	2.90	2.96	2.98	2.94	3.02	2.90	2.86	2.93	2.87	2.83	2.91	2.87	2.83	2.91	2.88	2.84	2.93
	Perceived severity Ebola*	4.69	4.66	4.71	4.75	4.73	4.78	4.73	4.70	4.75	4.73	4.71	4.75	4.74	4.71	4.76	4.71	4.69	4.74
	Concerns about own health	2.78	2.75	2.82	3.23	3.19	3.26	3.34	3.30	3.37	3.26	3.23	3.30	3.18	3.15	3.22	3.14	3.11	3.18
	Concerns about health family members	3.03	3.00	3.07	3.66	3.63	3.70	3.80	3.77	3.83	3.72	3.68	3.75	3.61	3.58	3.65	3.56	3.52	3.59
Perceptions of the measures against SARS-CoV-2	Sufficient measures are taken	3.57	3.54	3.61	4.05	4.01	4.08	3.86	3.83	3.90	4.07	4.04	4.10	4.09	4.06	4.13	3.85	3.81	3.88
	Measures are effective†	.	.	.	.	.	.	4.28	4.25	4.30	4.39	4.36	4.41	4.39	4.36	4.42	4.23	4.19	4.26
	Most others adhere to measures†	.	.	.	.	.	.	4.25	4.22	4.28	4.15	4.12	4.18	4.00	3.97	4.03	3.90	3.87	3.93
	Difficult to adhere to measures†	.	.	.	.	.	.	1.98	1.93	2.02	2.12	2.08	2.16	2.35	2.31	2.40	2.32	2.27	2.36
Trust in authorities	Trust information RIVM†	.	.	.	.	.	.	4.18	4.14	4.21	4.24	4.21	4.27	4.14	4.11	4.17	4.14	4.11	4.17
	Trust measures government†	.	.	.	.	.	.	4.06	4.03	4.09	4.15	4.12	4.18	4.10	4.07	4.13	4.01	3.98	4.04
Survey topic	Variable	%	95% CI		%	95% CI		%	95% CI		%	95% CI		%	95% CI		%	95% CI	
Self-reported protective behavior	Self-reported protective measures taken	17.04	15.70	18.40	79.16	77.50	80.70	87.69	86.40	88.90	84.83	83.40	86.20	81.24	79.70	82.70	79.57	78.00	81.10
	Self-reported adherence to guidelines†	.	.	.	.	.	.	93.96	93.00	94.80	91.59	90.50	92.60	87.64	86.30	88.90	85.47	84.10	86.80

*These variables are not part of the perceptions of COVID-19, but serve as comparison for the variable Perceived Severity COVID-19.
†These variables were not measured on T1 (24 Feb – 1 Mar) and T2 (16-23 Mar).

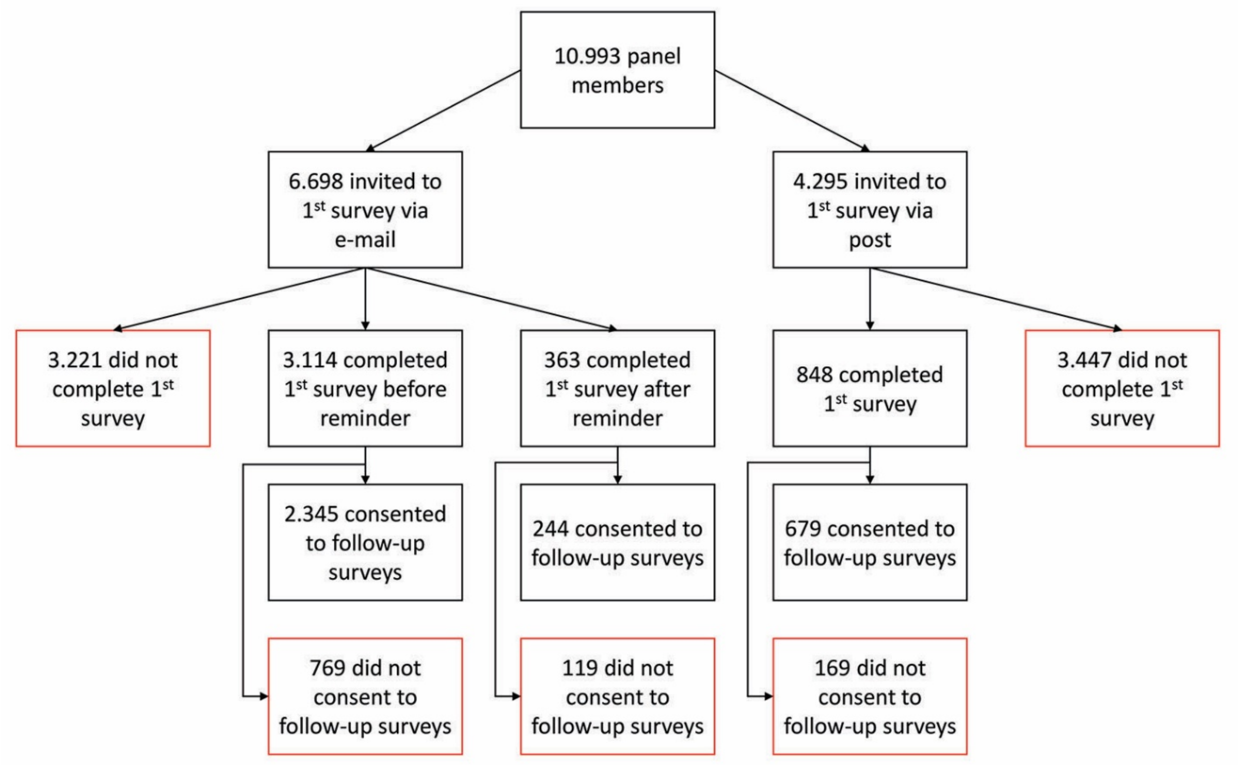

Appendix Figure. Overview of the response rate for the first survey per invitation route (e-mail/post), and the proportion of respondents who consented to be invited for the follow-up weekly surveys.
